# A genderful research world: rapid review, design, and pilot study of an interactive platform for curated sex and gender health research resources

**DOI:** 10.1186/s12939-023-01899-2

**Published:** 2023-06-20

**Authors:** Irene Göttgens, Jasmijn A.M. Sleutjes, Katelynn E. Boerner, Lena D. Sialino, Natália Valdrighi

**Affiliations:** 1 Department of Primary and Community Care, Radboud University Medical Center, Nijmegen, the Netherlands; 2 Department of Gastroenterology and Hepatology, Erasmus MC, Rotterdam, the Netherlands; 3 Department of Pediatrics, BC Children’s Hospital Research Institute, University of British Columbia, Vancouver, BC Canada; 4 Department of Health Sciences, Faculty of Science, Amsterdam Public Health Research Institute, Vrije Universiteit Amsterdam, Amsterdam, the Netherlands; 5 Department of Experimental Rheumatology, Radboud University Medical Center, Nijmegen, the Netherlands

**Keywords:** Sex, Gender, Research methods, Information dissemination, Knowledge mobilization

## Abstract

**Background:**

Integration of sex and gender into health research is best practice for designing and conducting equitable, rigorous scientific research. Many evidence-based resources exist to support researchers in this endeavour, but such resources often remain underutilized as they are difficult to find, are not publicly accessible, or are specific to a particular research phase, context, or population. The development and evaluation of a repository of resources was deemed important to create an accessible platform for promoting sex- and gender-integration in health research.

**Methods:**

A rapid review was conducted of critical resources for conducting sex and gender health research. These were integrated into a prototype website design (the *Genderful Research World*; GRW) that provided an interactive digital landscape for researchers to access these resources. A pilot study evaluated the GRW website for applicability, desirability, and usability with an international sample of 31 health researchers from various disciplines and career stages. Quantitative data from the pilot study was summarized with descriptive statistics. Qualitative data was summarized narratively and used to identify concrete elements for improvement in a second design iteration.

**Results:**

Results of the pilot study revealed that the GRW was considered user friendly and desirable by health researchers and helped them access relevant information. Feedback suggested that providing these resources in a playful way may enhance the experience of the user, particularly given the high ‘desirability’ scores and that users emphasized the interactive layout as being key to their intention to integrate it into their teaching endeavors. Key feedback from the pilot study (e.g., addition of resources specific to research with transgender populations, revision of website layout) was integrated into the current version of the website: www.genderfulresearchworld.com.

**Conclusions:**

The present research suggests a utility for a repository of resources for integrating sex and gender considerations into research, and that providing a logical, intuitive means of cataloguing and navigating such resources is critical for usability. The results of this study may inform the development of other novel researcher-directed resource curation efforts to address health equity issues and encourage and support health researchers to integrate a sex and gender perspective in their work.

**Supplementary Information:**

The online version contains supplementary material available at 10.1186/s12939-023-01899-2.

## Background

Sex characteristics and gender dimensions have long been known to exert an influence on disease etiology, presentation, and treatment outcomes. Sex refers to biological characteristics that can differ between people who are male, female, or intersex; gender refers to social and cultural factors associated with living as a man, woman, or gender-diverse person, and can encompass a variety of expressions and identities. Sex- and gender-based analyses (often referred to as SGBA+) can inform research on disease mechanisms, development of new therapeutics, the gendered context of healthcare, as well as enhance scientific rigor and reproducibility.

Despite the evidence on the impact of sex and gender on health, research methods continue to neglect sex and gender considerations in biomedical, clinical, and public health research. Major disparities exist in the representation of girls, women, intersex, transgender, and gender-diverse people in health research. A survey of ten different health disciplines demonstrated that the majority of basic science research studies were conducted in male animals [1]. While sex and gender differences exist in pharmacokinetics and pharmacodynamics, the U.S. Food and Drug Administration does not compare dose and efficacy between men and women in phase II trials, and women are underrepresented in phase III clinical trials [2, 3]. Most studies on male-female differences are focused on simply conducting comparisons, attributed generally to sex characteristics, with little or no consideration of gender dimensions. The small amount of research that has been conducted on gender and health specifically has focused primarily on constructs of masculinity and femininity, often neglecting to situate these traits within a broader systems and sociocultural understanding of gender roles and how it intersects with other social positions of privilege and marginalization [4].

There are some signs of a shift towards more of a focus on SGBA + in the health sciences. Several granting agencies and governing bodies, including U.S. National Institutes of Health (NIH), Canadian Institutes of Health Research (CIHR) and the European Commission, have attempted to address the issue, with varying levels of implementation from encouragement to mandating SGBA + be addressed in funding applications [5–7]. Alongside this push, numerous authors have argued for the importance of SGBA + in health research, [8–17] as well as an increased focus on the gendered or sexist context of other aspects of the health research process (e.g., calls to end all-male conference panels, encouragement of gender equity in research leadership and administration, gender biases in funding, etc.) [18–20].

To support increased awareness and attention to SGBA + in health research, many initiatives and key resources exist to support researchers, including checklists, fact sheets, and training modules [21]. However, such resources can be hard to find, busy and overwhelming, and many resources are specific to a particular research phase, population, or context. As a result, finding relevant resources can be time consuming and many resources are too specific or general for researchers’ particular needs. McGregor and colleagues conducted a review of resources related to incorporating sex and gender into medical research, in response to the NIH requirement for all grant applications to address the role of sex as a biological variable in vertebrate animal and human studies [22]. This review, which focused on literature published through 2014, identified 38 resources that met their eligibility criteria. While this allowed for the compilation of a useful database, many of the included resources are not available open-access, and many are currently more than 10 years old. In a field that is rapidly shifting, there is a clear need to explore innovative ways of providing updated, streamlined, and accessible reviews of the available SGBA + resources.

To address this aim, the Genderful Research World (GRW) was conceptualized. The research team was established as part of the ZonMw Gender in Health research fellowship and comprised an international multidisciplinary group of doctoral and postdoctoral researchers and clinician-scientists with an interest in SGBA + in health. The GRW platform was developed with the goal to support scientists in health and biomedical research who are looking for resources on how to integrate sex and gender into their work. This manuscript describes the development and pilot testing of the web based interactive GRW platform.

The aim of this study was to assess the applicability, desirability, and usability of the Genderful Research World interactive platform prototype amongst an audience of international health researchers. Secondary aims include an examination of participant-reported feedback, and a description of how this feedback was incorporated in an iterative fashion into the existing platform.

## Methods

A rapid review was first conducted to identify and select core resources for inclusion in the platform. These resources were incorporated into an initial GRW website prototype that was designed by the research team, with the support of a professional web developer. The initial GRW was pilot tested with a group of international health researchers, who provided feedback on design and implementation of this resource. This pilot study draws on quantitative and qualitative data collected by means of a digital questionnaire from December 2021 to March 2022. The study was non-WMO approved by the ethical committee of the VU medical center. The present reporting was informed by Bazzano and colleagues’ guideline for health research involving design [23].

### Identification of relevant resources: rapid review

A complete protocol for the rapid review is available in the Supplementary Material as Appendix A. The aim of the review was to identify existing resources that fit into the envisioned structure of the website: resources presented separately for pre-clinical and clinical/public health research pathways, covering each phase of the research process (e.g., research design, applying for funding, data collection, etc.).

First, two key existing websites by established leaders in SGBA + in health research, CIHR Institute for Gender and Health (CIHR-IGH; [24]) and the Gendered Innovations project [25], were reviewed and any relevant resources were extracted. The websites were searched page by page by a single reviewer, including opening any links on the pages. Resources were excluded if they were specific to the institute (e.g., descriptions of funding opportunities), were not related to sex and gender, or would not have broad applicability (e.g., resources about sex differences in a particular disease type, though some of these resources were retained to potentially include as case examples). An attempt was made to locate at least 5–7 resources for each research phase, with representation of both pre-clinical and clinical research.

Once both websites had been thoroughly searched and the eligible resources categorized into the identified research phases, hand-searching was conducted by one researcher (K.E.B.) using Google to identify additional resources that would address the outstanding research phases that did not have sufficient resources identified. Researchers also examined their own personal compilation of resources for any that might meet the criteria. One researcher (I.G.) compiled the final list of resources, attempting to capture a relatively equal distribution of resources from both the Gendered Innovations and CIHR-IGH websites (supplemented with others from hand-searching where applicable), selecting the resources that gave the most information with regards to the specific research phase upon opening the website. This criterion was to ensure that resources selected would have the most immediate value, rather than requiring the reader to scroll through too much content to get to the key messages. The results of the rapid review are available in Appendix B.

### Website development

The GRW website was established in collaboration with a professional web developer, using GitHub platform and YMAL coding. The website was developed based on description of what the GRW intended to deliver, with the design inspired by a map of a nature landscape to encourage a sense of exploration and play, and to increase engagement. Two quizzes were built to trigger curiosity and reflection on SGBA + topics. After the quizzes, a Definition Door provided the user with options to select the specific Research Roads (biomedical and medical/health), leading to the different research phases from each road. The starting point for both Research Roads was the terminology and definitions of sex and gender and the general relevance of accounting for sex/gender in biomedical and health research. Other sections with resources on “assembling research teams” and “applying for funding”, not specific to either research road, were also included.

Based on this script, a landscape map was designed to provide users with an overview of the website and the topics with sex and gender can and should be accounted for. To improve user experience, multiple ways of exploring the website were included: via the interactive map and the top menu, with an option for directly search for resources without going through the landscape (Literature Lake). Instructions on how to navigate the website were also provided. The most current version of the website is demonstrated with still images in Appendix C.

### Feasibility/usability testing

**Recruitment and eligibility.** Participants were recruited through the ZonMW Gender in Health Fellowship network and informal networks of the research team. Purposive sampling was used to target a range of researchers at varying career stage, familiarity with sex and gender research, and discipline. Individuals were eligible to participate if they met the following criteria and provided consent: (a) Researcher (including trainees) currently working in the field of biomedical, clinical, or public health research; (b) Age 18–75 years; (c) Have access to a computer or mobile phone with internet; and (d) Able to read and write in English.

**Procedure.** Potential participants completed an online informed consent page before entering in the interactive parts of the GRW. After browsing the website and available resources, participants were asked to complete a series of questionnaires regarding their perceptions of the GRW platform.

**Measures.** Participant demographics included age, sex, gender, country of origin and residence, career level, research field, and area of expertise. Participants indicated what information on SGBA + they were looking for, and the research phase of specific interest. A total of 29 items were included in the investigator-designed survey (see Appendix D) to evaluate the feasibility of the GRW platform across three domains: applicability, usability, and desirability. Items were rated on a 5-point Likert scale, and key targets/questions were informed by existing resources on the development/evaluation of innovations [26–28]. Applicability was defined as the quality of GRW being relevant or appropriate for its purpose, usability as ease of use for specific users in different settings, and desirability as the added value and innovation of the GRW platform.

**Data analysis.** Quantitative data was summarized with descriptive statistics. While the sample size was too small to permit comparisons between groups, descriptive trends were examined based on participant career level, identified expertise in sex/gender integration in research, and primary field of research. Qualitative data was also summarized narratively and used to identify concrete elements for improvement in a second design iteration and create an informative overview of the explanation of the quantitative scores. Illustrative quotes presented in the results.

## Results

**Demographics.** The final sample of participants included 31 international health researchers; complete demographic information is presented in Table 1. Most of the sample identified as women, primarily in early adulthood and at the junior/PhD-level stage. Europe was over-represented as both the region of origin and current residence, in particular, the Netherlands. Most researchers identified their primary area as clinical or public health research. Most participants (*n =* 28, 90%) accessed the website from a computer (laptop or desktop) rather than a mobile device.

**Table 1:** Frequency and percentage of participant demographics, split by how often participants provided free-text feedback

	Whole study population *n* = 31	Provided feedback often ^c^ *n* = 12	Provided feedback less often ^c^*n* = 19
**Gender Identity** ^**a**^			
Woman	27 (87%)	9 (75%)	18 (95%)
Man	3 (10%)	2 (17%)	1 (5%)
Non-binary	1 (3%)	1 (8%)	-
**Age**			
18–29 years	17 (54%)	6 (50%)	11 (58%)
30–39 years	7 (23%)	2 (17%)	5 (26%)
40–49 years	5 (16%)	2 (17%)	3 (16%)
50–59 years	2 (6%)	2 (17%)	-
**Region of Origin**			
Australia	1 (3%)	1 (8%)	-
Europe	26 (84%)	9 (76%)	17 (90%)
North America	3 (10%)	2 (16%)	1 (5%)
South America	1 (3%)	-	1 (5%)
**Region of Current Residence**			
Australia	1 (3%)	1 (8%)	-
Europe	25 (81%)	8 (74%)	16 (74%)
North America	4 (13%)	2 (16%)	2 (11%)
South America	1 (3%)	-	1 (5%)
**Career level**			
Junior / PhD	19 (61%)	8 (67%)	11 (58%)
Mid-level / Post-doc	4 (13%)	-	4 (21%)
Senior / Professor	8 (26%)	4 (33%)	4 (21%)
**Research area** ^**b**^			
Preclinical / basic	5 (16%)	2 (17%)	3 (16%)
Clinical	10 (32%)	4 (33%)	5 (26%)
Public health	18 (58%)	6 (50%)	11 (58%)
**Self-reported sex/gender expertise**	7 (23%)	2 (17%)	5 (26%)
**Topic participants were most interested in accessing sex/gender resources about** ^**b**^			
Assembling research teams	7 (23%)		
Applying for funding	9 (39%)		
Preclinical/basic research	9 (39%)		
Clinical/ public health research	24 (77%)		
**Research phases participants were most interested in accessing sex/gender resources about** ^**b**^			
Funding	17 (55%)		
Study population and sample size	23 (74%)		
Research question & design	21 (68%)		
Data collection and follow-up	18 (58%)		
Data analysis	19 (61%)		
Interpretation and translation	26 (84%)		

*Note.*^a^ Gender identity was elicited in a free-text box, responses that were listed as “female” or “she” were coded as “woman”, and responses listed as “male” were coded as “man.” ^b^ Participants could select more than one option; therefore, the total may not add up to 100%. ^c^ Feedback provided “often” was defined as the participant providing free-text feedback on at least 20% of the questions, “less” often feedback was defined as less than 20% (note that 2 participants did not provide any free-text feedback). Note that this split is not reported for topic and research phase of most interest, as the amount of free-text feedback was determined to be less relevant for these areas

***Experience with and interest in sex/gender research.*** The majority (*n* = 24, 77%) of the participants in our study identified as non-experts in sex/gender research. Congruent with the primary research area of participants, most identified being interested in accessing resources related to sex and gender in clinical/public health research (*n* = 24, 77%), but some also identified issues regarding sex and gender as it relates to building research teams and applying for funding as being relevant to their needs. When asked what phases of the research cycle specifically that participants were interested in accessing resources about, the most common was interpretation and translation of study results (*n* = 26, 84%), followed by study population and sample size, and research question and design.

**Website feedback.** Summarized below is the feedback for each area investigated, as well as how the feedback was incorporated into a revised version of the website. Descriptive statistics for the quantitative ratings of the website are presented in Table 2, and integrated below are illustrative quotes from the free-text feedback provided by participants. Nearly all (n = 29, 94%) participants provided free-text feedback on at least one of the questions, with *n =* 13 (42%) providing feedback on at least 20% of the questions. All of the individuals who identified themselves as sex/gender experts provided at least some feedback.

**Table 2:** Quantitative results for applicability, usability, and desirability questionnaire by career level, sex/gender expertise, and research field

		Career level			Expertise in sex and gender health research		Research field		
	Total (*n* = 31)	Junior (*n = 19*)	Mid (*n = 4*)	Senior (*n = 8*)	Yes (*n = 7)*	No (*n = 24*)	Pre-clin/basic (*n = 5*)	Clinical (*n = 19*)	Public health (*n = 17*)
**Applicability**									
A1 (quiz triggered knowledge)	4.2	4.2	4.3	4.2	**3.6**	4.3	**3.8**	4.3	4.2
A2 (quiz made me think)	4.0	4.0	4.3	3.9	**3.3**	4.2	4.0	4.3	3.8
A3 (easy to move through quiz)	4.5	4.3	4.5	4.8	4.3	4.5	4.0	4.6	4.5
A4 (organization resources good)	4.0	**3.9**	4.0	4.0	4.0	4.0	**3.8**	4.2	3.9
A5 (research phases comprehensive)	4.3	4.3	4.0	4.3	4.3	4.3	4.4	4.2	4.2
A6 (useful resources)	4.0	**3.9**	4.0	4.1	4.3	**3.9**	**3.8**	4.0	4.0
A7 (resources new)	**3.8**	**3.8**	4.0	**3.5**	**3.1**	4.0	4.2	4.2	**3.4**
A8 (resources enough information)	4.0	4.0	4.3	4.0	4.3	4.0	**3.9**	4.0	4.2
A9 (overall applicability)	7.8	7.7	7.3	8.4	8.0	7.8	7.8	8.1	7.7
**Usability**									
U1 (access quick and intuitive)	4.3	4.3	**3.8**	4.4	4.3	4.3	4.8	4.2	4.2
U2 (website easy on eyes)	4.0	**3.9**	4.0	4.1	4.3	**3.9**	4.6	4.1	**3.8**
U3 (start instructions clear)	**3.8**	**3.7**	**3.5**	4.0	4.4	**3.6**	4.2	**3.4**	**3.8**
U4 (quickly found information)	**3.5**	**3.5**	**3.8**	**3.6**	**3.3**	**3.6**	4.4	**3.4**	**3.4**
U5 (became familiar quickly)	4.2	4.2	4.0	4.4	4.3	4.2	4.4	4.2	4.1
U6 (easy to use)	4.4	4.4	4.0	4.5	4.1	4.4	5.0	4.3	4.2
U7 (functions well integrated)	**3.9**	**3.8**	**3.8**	4.1	4.3	**3.8**	4.0	4.0	**3.9**
U8 (information text clear)	4.0	**3.9**	4.0	4.5	4.1	4.0	4.4	4.0	4.0
U9 (information text appropriate)	4.4	4.4	4.0	4.5	4.4	4.3	4.6	4.4	4.2
U10 (terminology understandable)	4.4	4.5	4.0	4.5	4.3	4.5	4.4	4.6	4.4
U11 (application device-friendly)	4.3	4.2	**3.8**	4.8	4.4	4.3	4.4	4.3	4.2
U12 (enjoyed use)	4.3	4.4	**3.5**	4.4	4.4	4.2	4.4	4.6	4.0
U13 (overall usability)	7.8	7.7	7.3	8.3	8.1	7.7	8.4	7.8	7.6
**Desirability**									
D1 (innovative)	4.3	4.3	4.5	4.2	4.3	4.3	4.4	4.4	4.3
D2 (interesting design)	4.2	4.1	4.3	4.2	4.0	4.3	4.4	4.6	**3.9**
D3 (fun presentation)	4.3	4.2	4.3	4.3	4.6	4.2	4.4	4.4	4.1
D4 (aesthetics improved experience)	4.1	4.1	**3.8**	4.2	4.3	4.1	4.4	4.2	4.0
D5 (functional for researcher)	4.3	4.1	4.5	4.5	4.0	4.3	4.2	4.2	4.3
D6 (matches preferences for searching)	4.0	**3.9**	4.0	4.2	**3.8**	4.0	**3.4**	4.3	4.0
D7 (feasible in time want to spend)	4.0	**3.9**	**3.8**	**3.3**	4.3	**3.9**	**3.8**	4.0	4.1
D8 (useful for all levels of experience)	4.0	**3.9**	4.3	4.7	4.3	**3.9**	**3.2**	4.1	4.2
D9 (relevant resource)	4.4	4.4	4.3	4.7	4.3	4.5	4.4	4.6	4.4
D10 (overall desirability)	8.3	8.2	8.3	8.8	8.1	8.4	8.4	8.4	8.3

*Note*. All questions were rated on a 1 (totally disagree) to 5 (totally agree) scale, except for the “overall” questions scored on a 0–10 scale. Scores lower than 4.0 are bolded to identify areas in need of improvement

***Applicability.*** The GRW website generally scored well with respect to applicability (7.8/10 overall), particularly among those at the Senior career level, and those who identified as having sex/gender expertise. As expected, these individuals rated the website lower in its ability to trigger their knowledge and encouraging them to think more extensively about sex/gender issues. Mid- career level and non-sex/gender-experts scored higher in GRW resources being new to them.

Free-text feedback (provided by 54% of participants) described the quiz prompting participants to think further about sex and gender integration in health research, though there was also concern regarding whether the quiz adequately captured the nuance of such complex concepts.

*“[The quiz] reaffirmed areas I understand and identified areas where I lack clarity.”* - Senior-level public health researcher (non-expert)

*“Many questions were vague or presented binary answers (true/false) for complex concepts that would depend on the context.”* - Junior-level clinical researcher (non-expert)

Participants also reported appreciating the extensiveness of resources, many of which were identified as being either new for the participants or linked to resources they already are familiar with and trust.

*“I would say there was sometimes even too much information for me to process. Therefore, I didn’t know where to click first sometimes. However, I can imagine that when you are looking for a particular topic, this is not a problem, and it is actually very useful that there are a lot of resources.”* -Mid-level public health researcher (non-expert)

While many described enjoying the design and visual appeal of the map, some reported room for improvement in the organization of the map layout. Feedback was also provided regarding the need for more information on addressing gender diversity in research:

*“Does not include adequate information or guidance for people who are either studying trans populations specifically or trying to include trans people in their research (as everyone should be doing).”* - Junior-level clinical researcher (non-expert).

Participant feedback led to revision of the quiz questions and resource layout, and addition of resources specific to conducting research with transgender populations.

***Usability.*** The GRW website was rated highly on usability (7.8/10 overall; complete scores presented in Table 2). Usability was rated as highest by Senior-level individuals, participants identifying as sex/gender experts, and pre-clinical/basic health researchers. Most researchers (particularly non-experts) identified room for improvement with respect to the websites’ instructions, navigating and finding information, and integrated functions. Mid-level researchers scored the website lower on device-friendliness and reported less enjoyment of the website than other groups.

Feedback on the free-text questions (answered by 79% of respondents) described the website as providing a helpful overview of sex/gender research resources. They reported the separation by research phase to be a logical and intuitive way of navigating the website.

*“Plain and simple, but very informative.”* - Senior-level pre-clinical researcher (non-expert).

*“Logical layout and easy to navigate.”* - Senior-level public health researcher (non-expert).

However, suggestions were offered to improve readability and accessibility with respect to text and general navigation.

*“You shouldn’t need instructions to understand the website. Maybe it can be integrated in the map (e.g., using arrows for start and endpoint).”* - Junior-level clinical researcher (non-expert)

As such, the GRW website underwent revisions to make the starting point clearer, improve the instructions, undergo a thorough English-language check, and integrate a search function. Inactive links were also replaced.

***Desirability.*** Participants described the GRW website as useful across all levels of experience, and the website scored highest on desirability of the 3 domains assessed (8.3/10 overall). Junior and pre-clinical science health researchers were more likely to report that this format did not represent their preferred method of searching for information. The lowest desirability score was with respect to whether GRW represented a feasible option for finding resources in the time they have to spend on this activity, perhaps reflecting a need for a more concise alternative.

Many (61%) participants provided free-text feedback on desirability. Participants reported this was a novel way to present resources and they found the format fun and easy to use.

*“I think the map is very novel compared to a website that just provides several resources. It makes it more interesting and therefore more fun to do (...) It may take a couple of minutes to learn how to navigate the site, but information on the site is easier to find than a google search.”* - Junior-level public health researcher (expert).

*“(…) it is an easy oversight into a complex field.”* - Junior-level public health researcher (non-expert).

Some participants also referenced plans for future use:

*“I am going to use it, even in my teachings.”* - Junior-level clinical and public health researcher (expert).

*“I would definitely return to this resource, especially when writing grant/scholarship applications!”* - Junior-level clinical researcher (non-expert)

*“(...) all in 1 place, divided up in usable categories to make it easy to find exactly what you are looking for. I’m bookmarking this page!”* - Senior-level clinical researcher (non-expert)

Participant feedback identified that there was some room for improvement with respect to the layout of the page, which led to changes to increase consistency across pages on the website.

## Discussion

This manuscript describes the development and pilot testing of a novel website (www.genderfulresearchworld.com) intended to consolidate existing sex and gender resources in a playful and interactive way to health researchers. The rapid review identified 45 pre-existing resources across a range of research phases and topics, relevant to pre-clinical and clinical/public health researchers. These results were integrated in a web-based platform and feasibility testing showed that health researchers rated the resource website as applicable, usable, and desirable. Qualitative feedback from participants exhibited an enjoyment of the visual aspects of the platform, an intention to revisit the website for future use, and provided critical feedback that was integrated into a new design iteration, which resulted in the current version of the website.

A strength of this research was the pragmatic utilization of existing resources, and the development of the website by a diverse group of researchers with respect to research discipline, expertise, and nationality. This allowed for rich discussions of the applicability of this work to a variety of contexts, which is reflected in the website design and content. The inclusion of stakeholder feedback in iterating on the website design is also reflective of best practices in inclusive design processes, though future iterations would benefit from increased diversity amongst stakeholders. While a wealth of literature is available to support the development and dissemination of resources, little is available to guide the construction of such a meta-resource. Further work in this area may be warranted, particularly given the challenges faced by the present authorship team, namely, balancing pragmatism and completeness, navigating a rapidly changing landscape of available resources, determining how to select and distill available information, and making the website accessible and useful to a broad population of health researchers. The decision to base the present rapid review primarily on two established resources (CIHR Institute for Gender and Health and the Gendered Innovations project) was made for pragmatic/feasibility purposes identifying first what resources were available from two existing, well-established, and reputable sources that were likely to be maintained. This was prioritized as our protocol did not involve an evaluation of the quality of evidence being included, a decision made to focus on knowledge dissemination rather than knowledge generation. This approach was supplemented by hand searching to fill gaps in resources, however, this approach did bias the content towards being heavily representative of these two primary resources, and other useful resources for integrating sex and gender in research may have been overlooked in the process.

Most participants indicated to be interested in accessing SGBA + resources related to interpreting and translating study results, as well as study population and determining sample size. These topics require more attention in the development of future SGBA + resources. Future research may also consider determining more specific issues that researchers encounter in applying a sex- and gender-based lens to their research during these stages; it may be that existing resources related to these topics are too general, as considerations vary greatly depending on whether the focus is on sex, gender, or both, the domain of science (e.g., basic vs. clinical), and the aim of the study (e.g., whether SGBA + is a primary focus) [29–31]. Additionally, while many existing resources are very practically focused on application of SGBA + to research design and analysis, participants thoughtfully raised that many of the existing resources represent an oversimplification of the complexity of sex and gender, and do not always address important and nuanced issues related to equity and representation in research (e.g., intersectionality, how to conduct research with gender-diverse or intersex populations, and how to situate SGBA + research findings within systemic and structural inequities in health) [4, 10, 32]. There is clearly a balance needed between general and field specific SGBA + resources, though much of the qualitative feedback suggested that the resources available were already more extensive and detailed than many participants had expected or would desire.

The present sample was reflective of the method of study recruitment in that many of the respondents were of a similar demographic to the study authors (primarily young women living in Europe), and recruiting within researcher’s existing networks may have increased the social desirability of responses. The sample was also limited with respect to representation from pre-clinical/basic research areas. As such, it is difficult to generalize as to whether the website would have received such favorable feedback among different target audiences, and results are likely more applicable to the clinically focused content of the website. However, it was encouraging that participants represented a range of career levels and levels of expertise in sex/gender research, allowing investigation the applicability of this resource across the career trajectory.

Much of the evaluation was focused on the GRW website itself, however, several insights can be drawn related to the broader sex and gender resource design and education context. Team assembly and study population/sample size were two areas where it was more challenging to find appropriate resources for inclusion in the rapid review. While less than a quarter of participants expressed an interest in SGBA + resources related to team assembly, this may have been reflective of the fact that most of our sample was comprised of junior researchers who may not have been at the stage of having responsibility for assembling research teams. Conversely, this may be an area of health research where less attention is paid to the importance of sex and gender, perhaps reflecting larger systemic gender biases and meritocracy beliefs [33, 34].

It is also worth noting that in the short period of time between when the rapid review was conducted (September 2021) and when the pilot study launched (December 2021), one of the resources linked in the webpage had already gone inactive. This highlights the challenge of such repositories and resources, the need for constant updating to reflect active resources and new information in a rapidly changing field. While the aim of this project was to generate data on user preferences that could inform future resource repository development, we have recommended to our funders, to whom ownership of the platform will be transferred following this pilot project, that updating and maintaining the GRW would be a worthwhile investment of resources. The results of this pilot also suggest that this approach be applied to other areas of research where there is a need to increase awareness and consolidate existing resources.

While the case for SGBA + has been made strongly in health research, and many resources are available, little research has considered the dissemination and gaps in existing resources. An existing review by McGregor and colleagues [22] found that insufficient data is available for evaluating the level of evidence upon SGBA + resources are based, and while many resources related to research design and methodology exist, not all are publicly available. The decision to consolidate pre-existing resources was made in part in reaction to the siloed nature of such resources. Many excellent resources are published in the form of peer-reviewed journal articles, which unfortunately often exist behind a paywall, making them inaccessible to researchers from low- and middle-income countries, as well as the general public [35, 36]. Creating a repository of open-access resources was intended to improve equity in health research, both in the respect of advocating for better SGBA + in research, but also increasing the ease of access to resources to support this more broadly. Additionally, much of the SGBA + research in health has centered on specific health problems or conditions that are known to have sex differences (e.g., cardiology, pain), or where there is a sex/gender-based controversy (e.g., the debate on whether sex differences exist in brain structure/function, or political debates regarding access to gender-affirming care for transgender and gender-diverse people). As such, many resources are targeted specifically to researchers working in these areas, an approach that may be too specialized to serve as introductory resource for more novel sex/gender researchers.

The increased attention to the inclusion of SGBA + in health research design and policy will inevitably necessitate many non-experts in sex and gender to find ways of integrating this perspective in their work. The intention of the Genderful Research World was to ease this process by having a one-stop shop for useful resources that researchers could access as a starting point, where they could select the resource relevant to the research stage/question they are hoping to address. The interactive and playful nature of the website was also intended to provide a positive, non-threatening experience, that would encourage researchers to come back, share, and continue to explore the ways they could integrate sex and gender into their research.

## Conclusions

The present research suggests a utility for a repository of resources for integrating sex and gender considerations into research, and that providing a logical, intuitive means of cataloguing and navigating such resources is critical for usability. Resources related to interpreting and translating study results, as well as study population and determining sample size were indicated to be of primary interest to health researchers. The present study also suggests that providing these resources in an interactive, playful way may enhance the experience of the user, particularly given the high desirability scores by participants. Despite the small sample size, participants provided substantial qualitative feedback for the iterative development of the platform and highlighted the critical importance of stakeholder partnerships in developing and implementing SGBA + resources.

## Electronic supplementary material

Below is the link to the electronic supplementary material.

Supplementary Material 1

Supplementary Material 2

Supplementary Material 3

Supplementary Material 4

## Data Availability

The authors confirm that the data supporting the findings of this study are available from the corresponding author (KEB) upon reasonable request.

## List of Abbreviations

CIHR-IGH: Canadian Institutes of Health Research Institute for Gender and Health
GRW: Genderful Research World
NIH: U.S. National Institutes of Health
SGBA +: Sex- and Gender-Based Analysis Plus
