# Supplementary figures and images for: A genderful research world: rapid review, design, and pilot study of an interactive platform for curated sex and gender health research resources
Source: Int J Equity Health. 2023 Jun 20;22:118. doi: 10.1186/s12939-023-01899-2 (PMC10283329; doi:10.1186/s12939-023-01899-2)

Appendix C: Screenshots of the GRW online platform


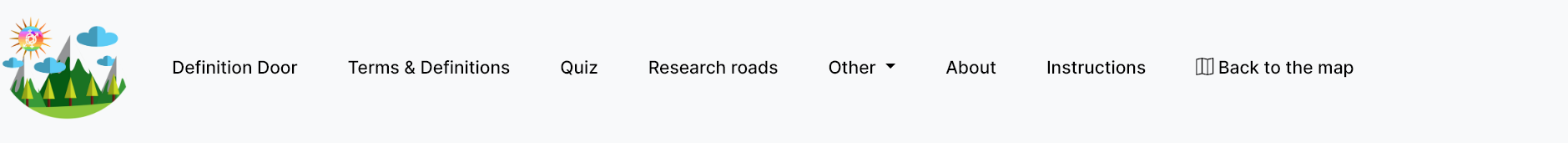


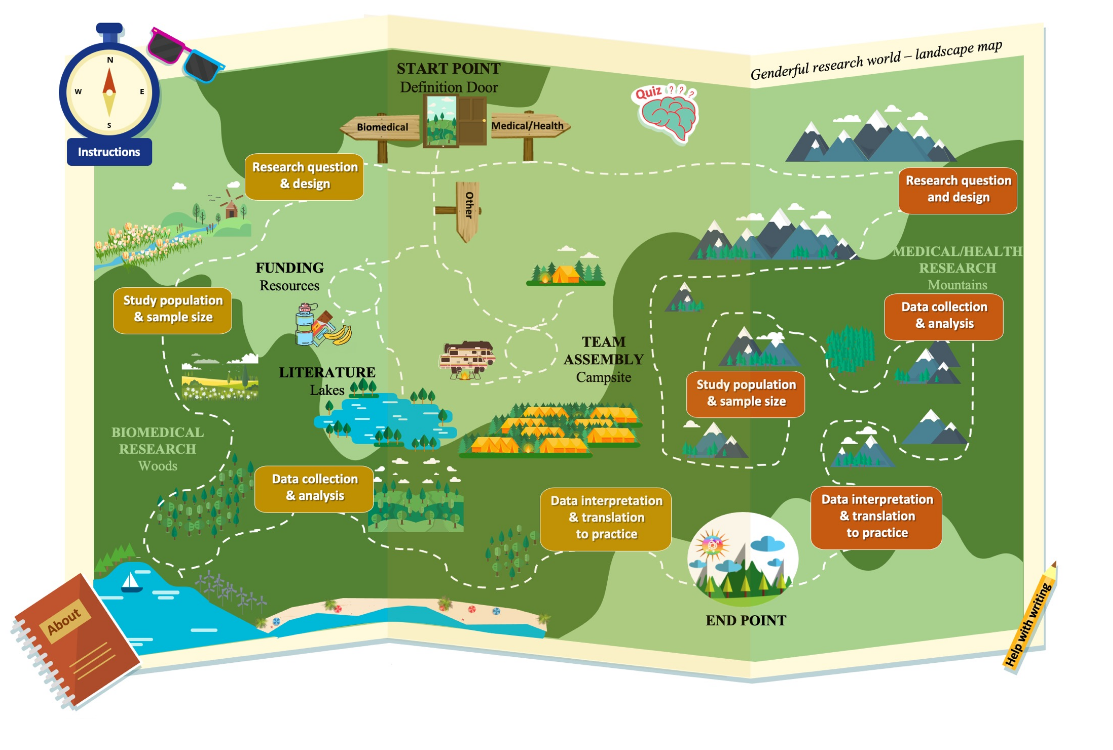


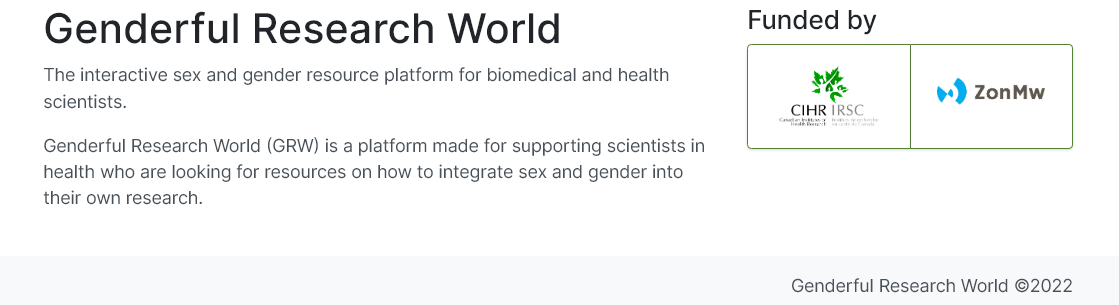


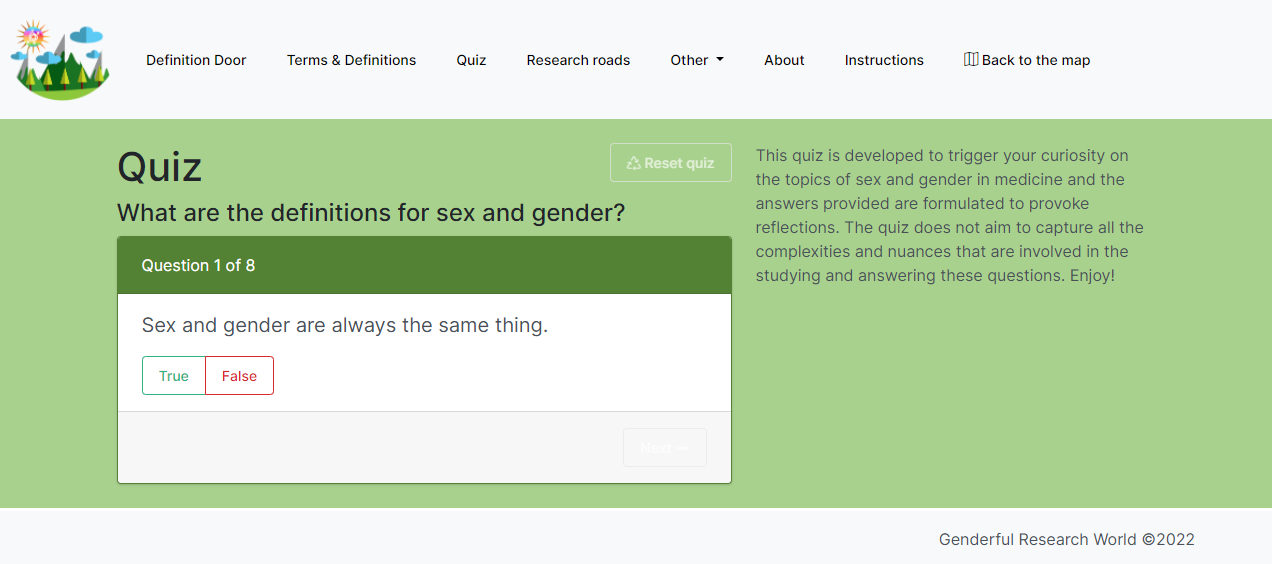

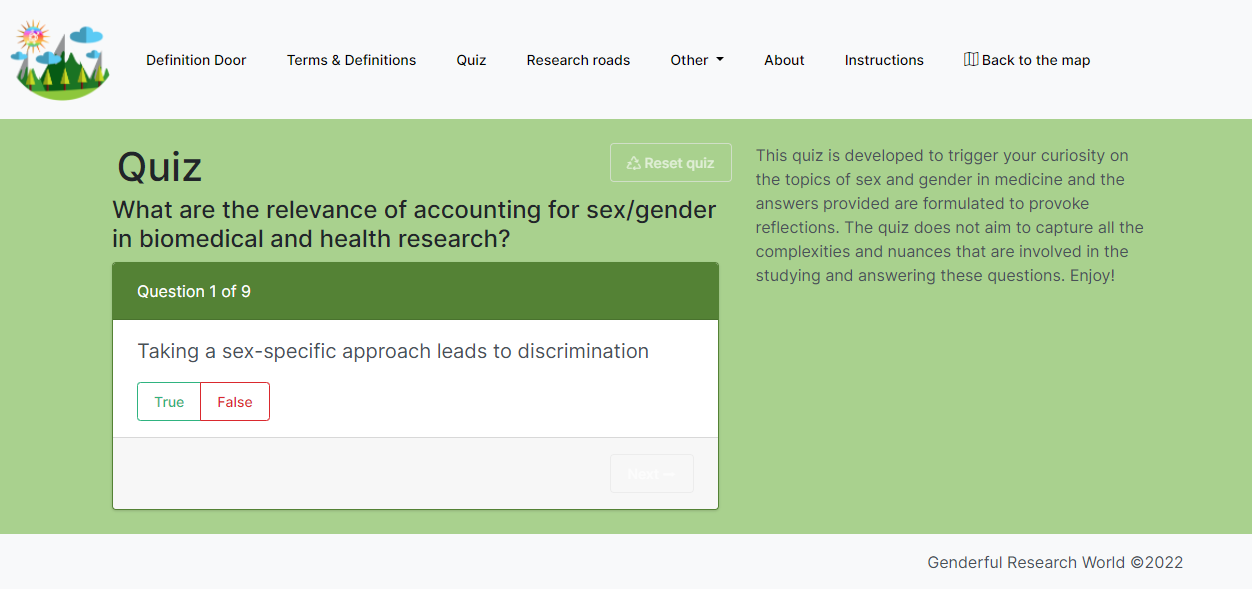


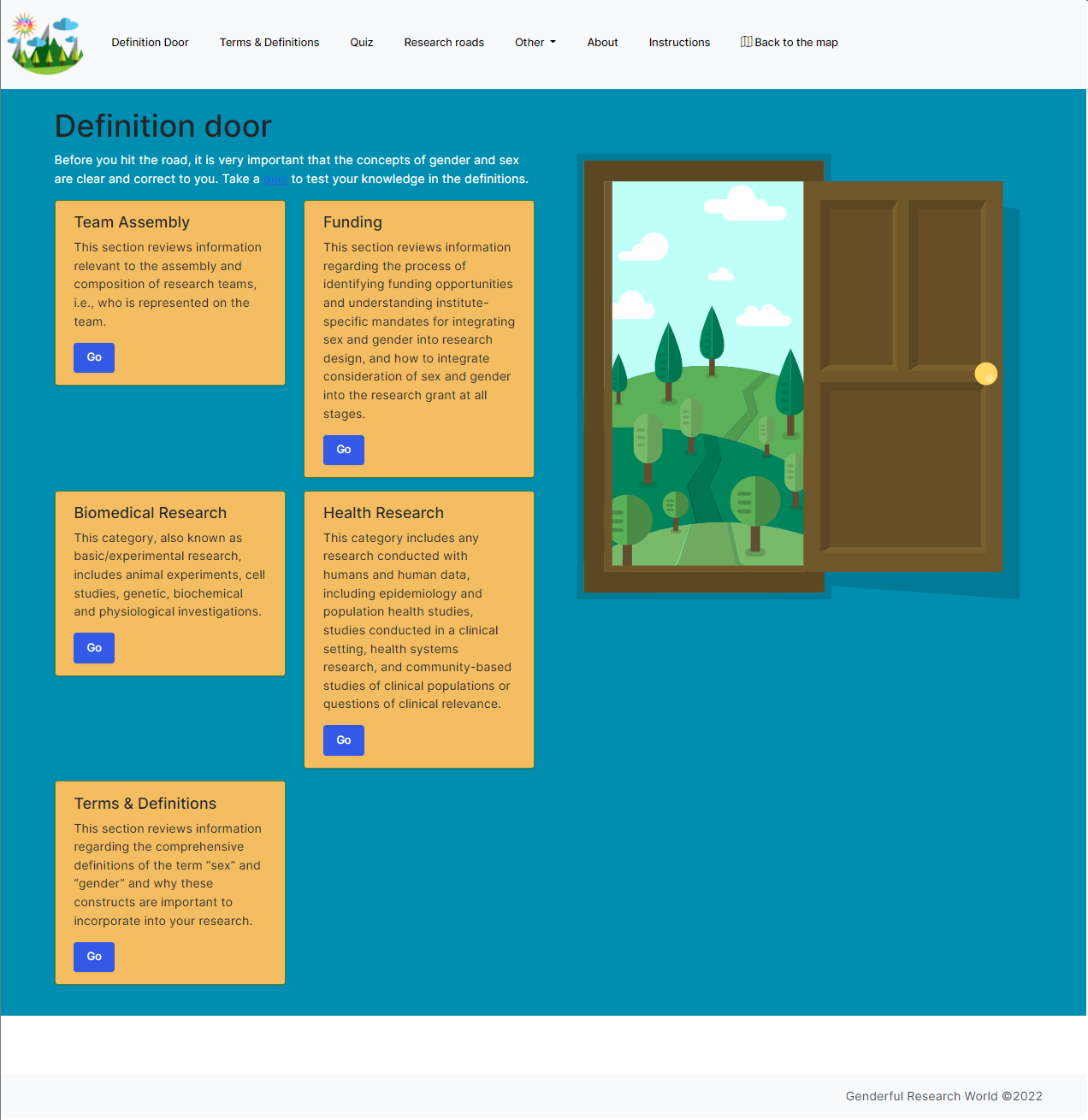


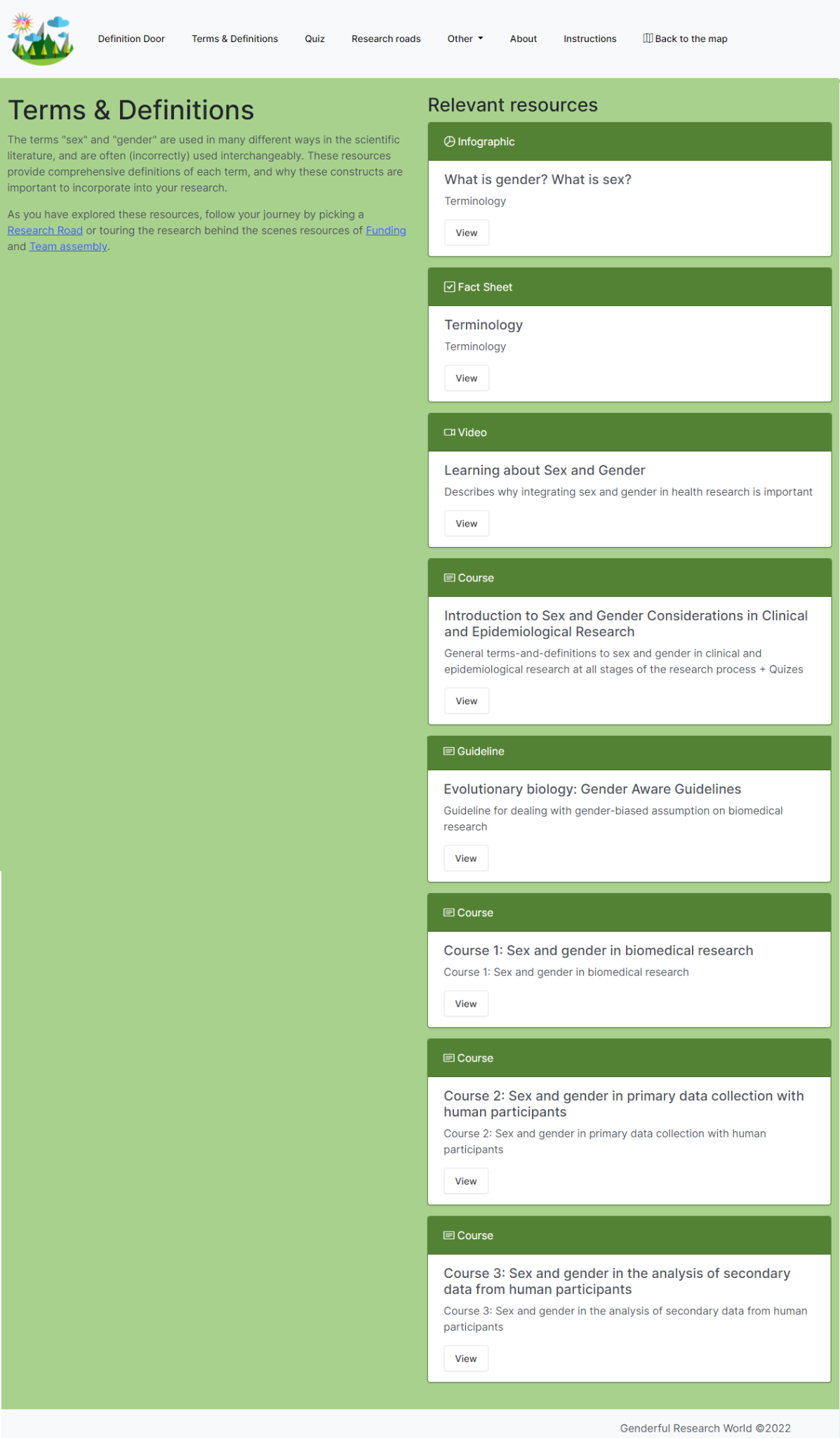


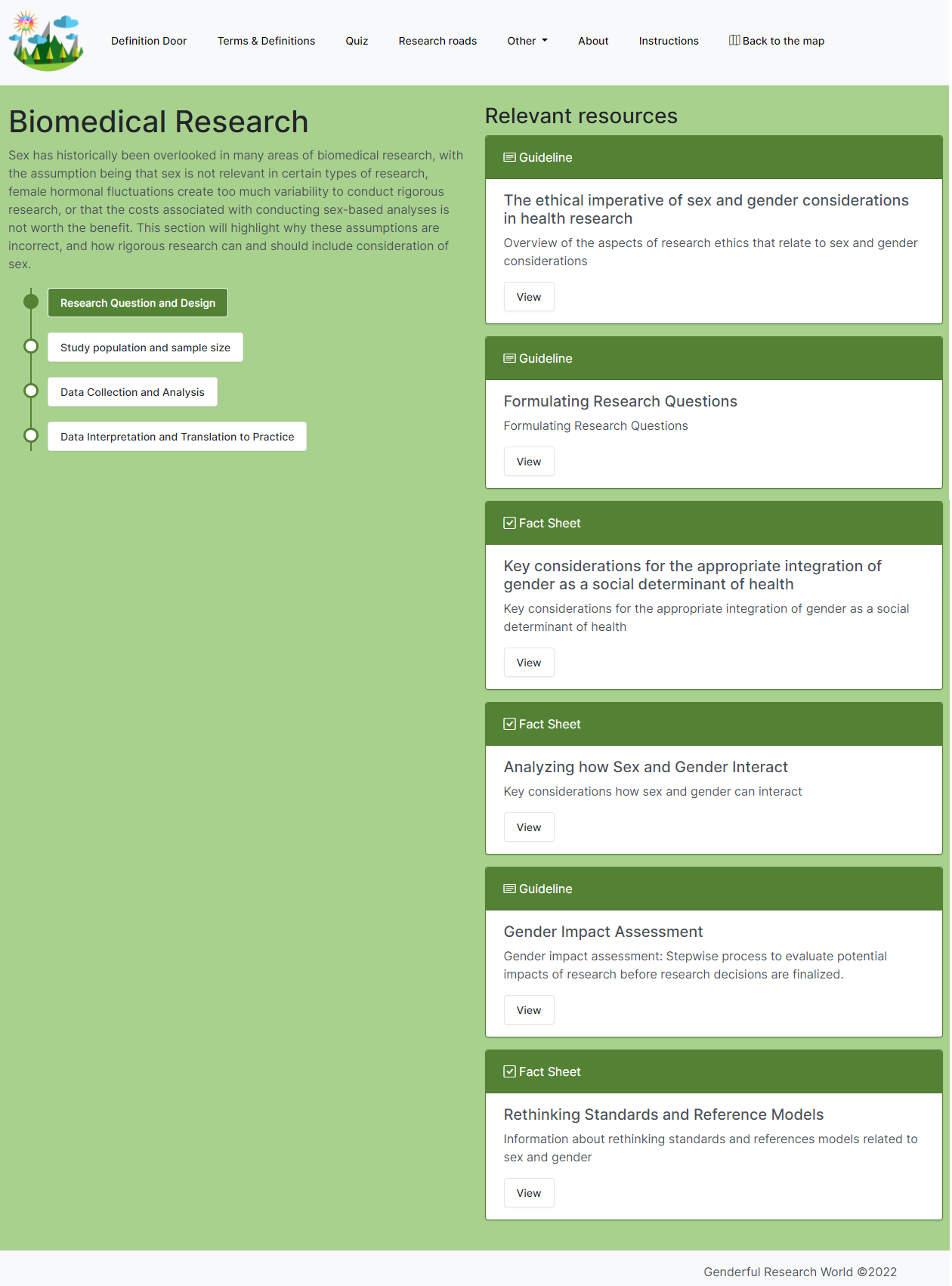

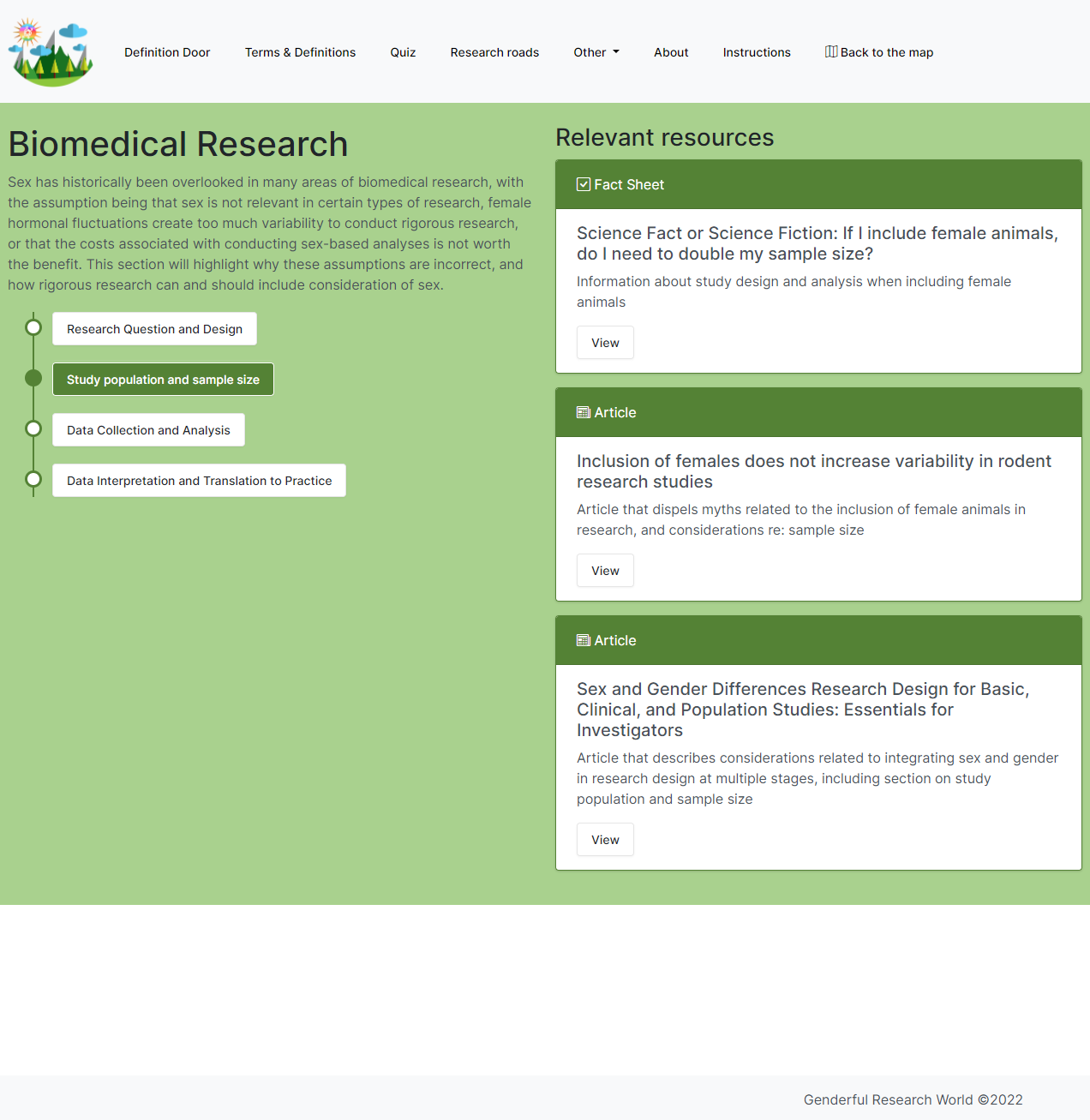

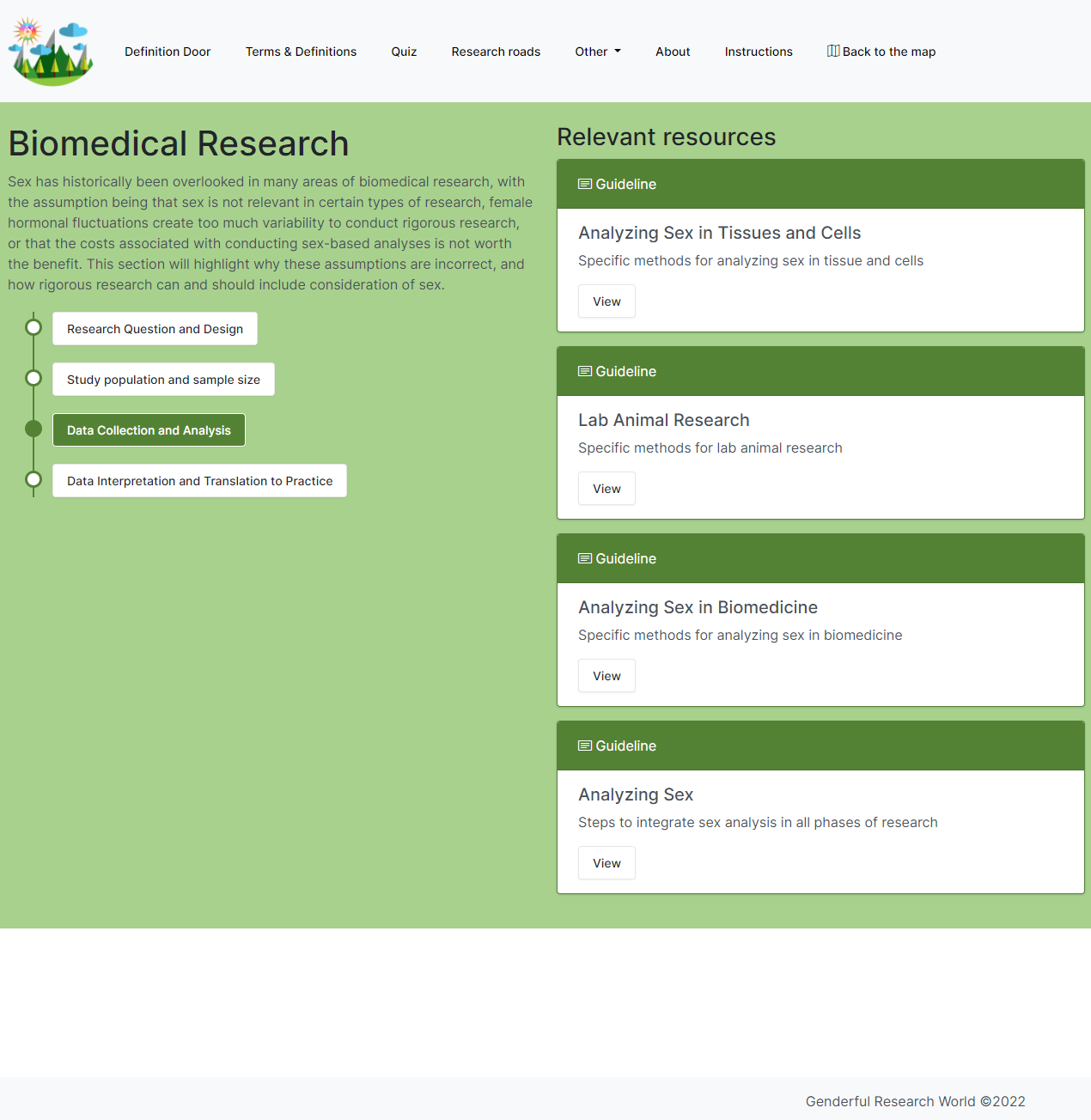

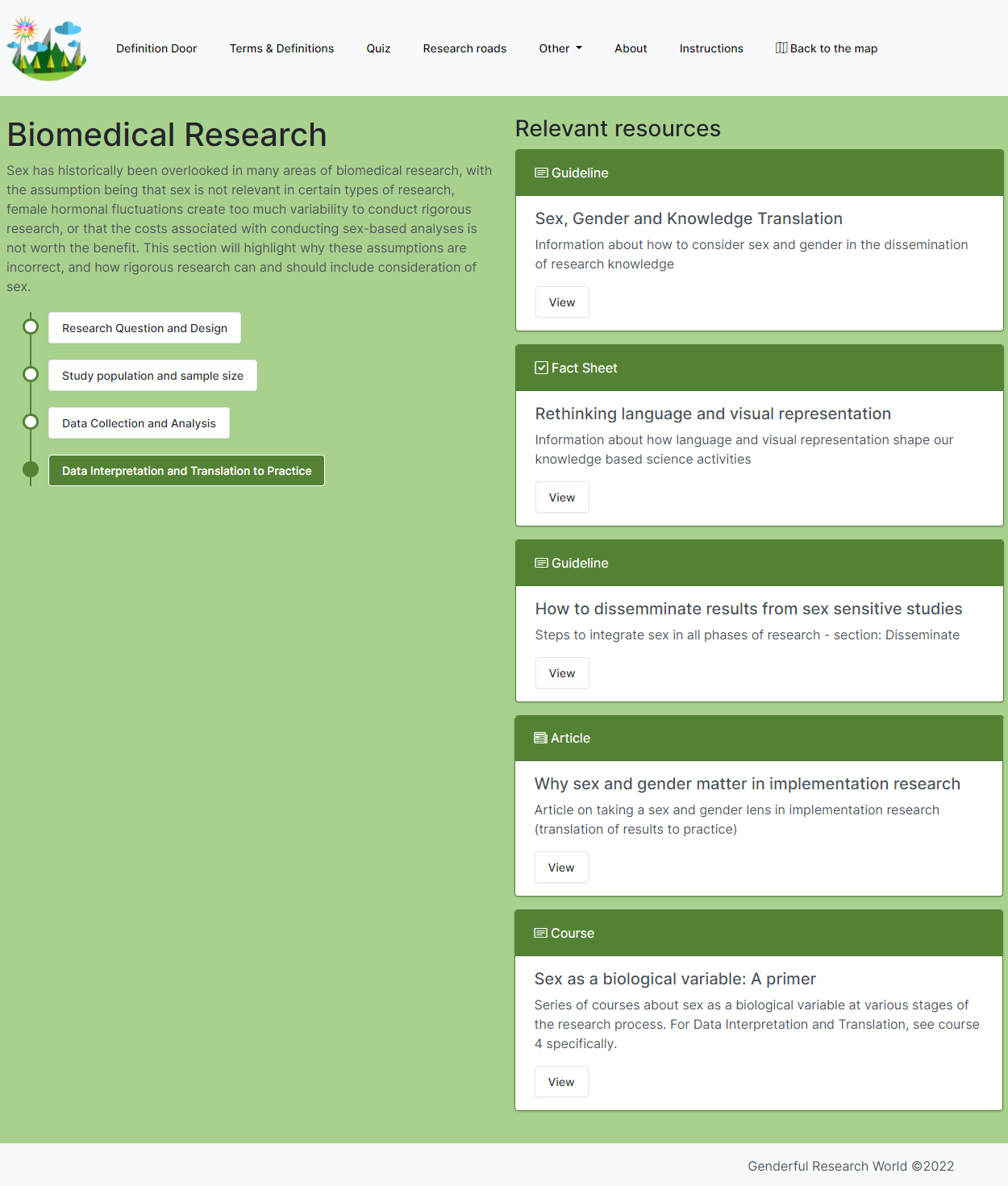


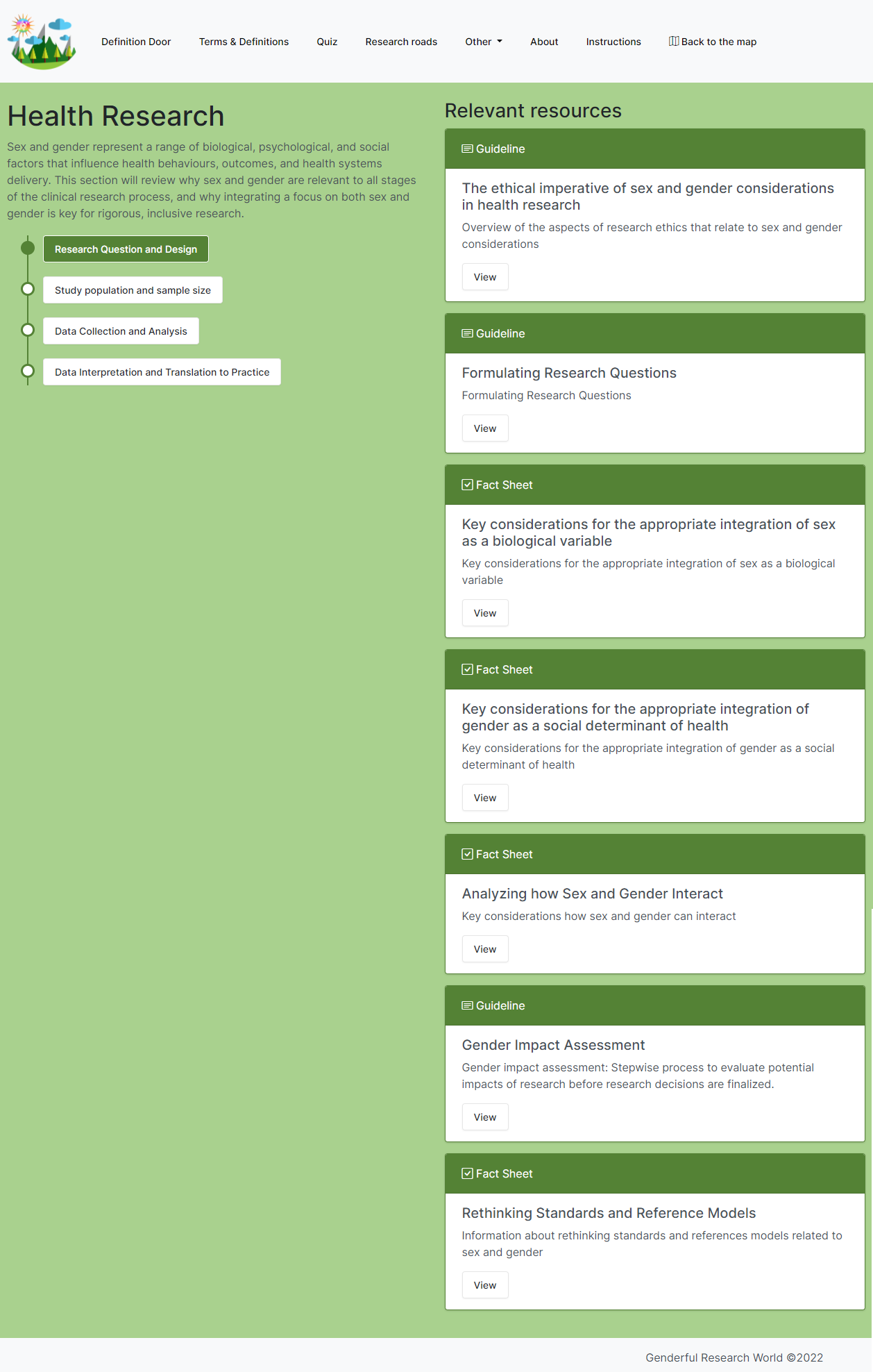

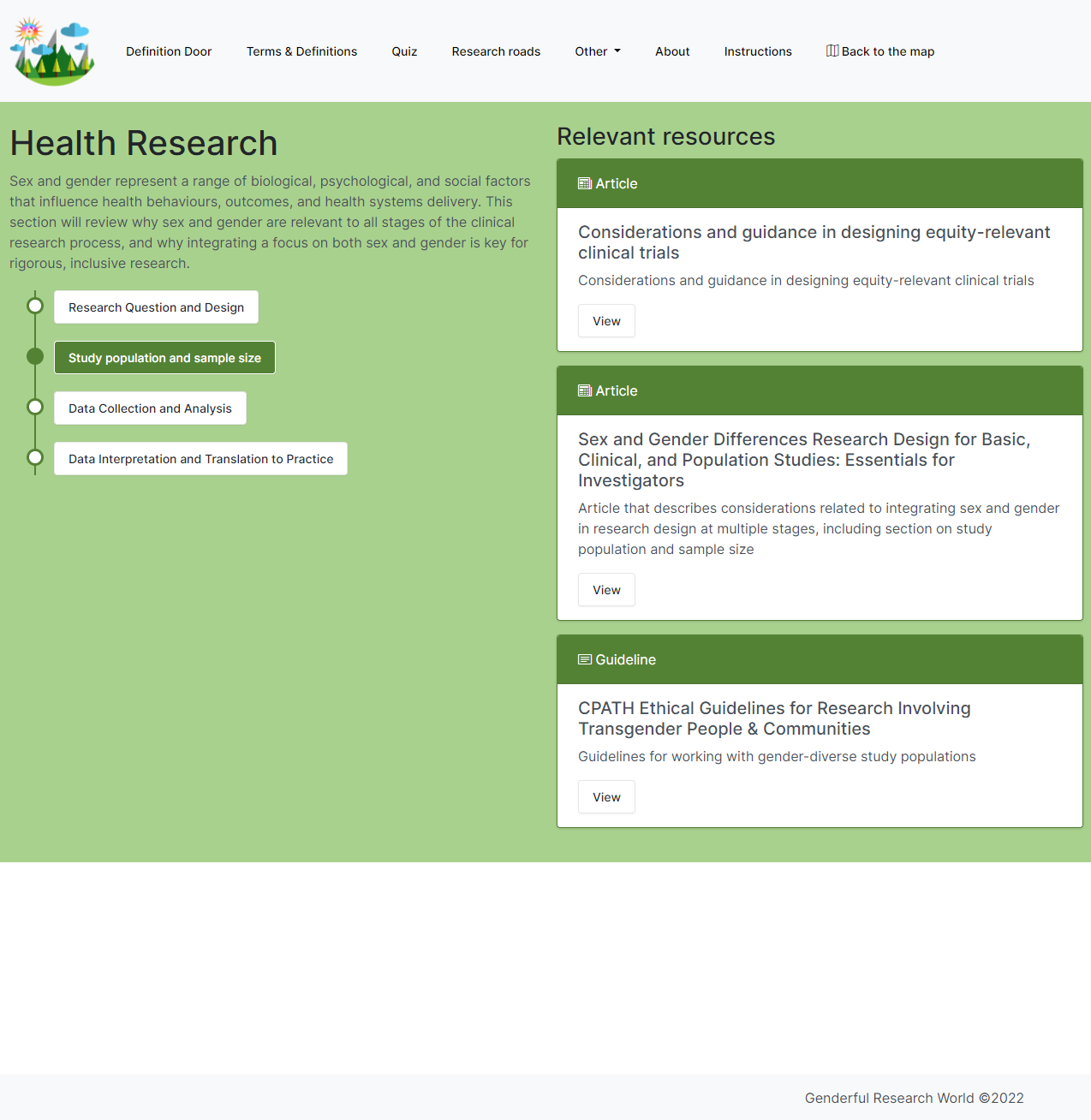

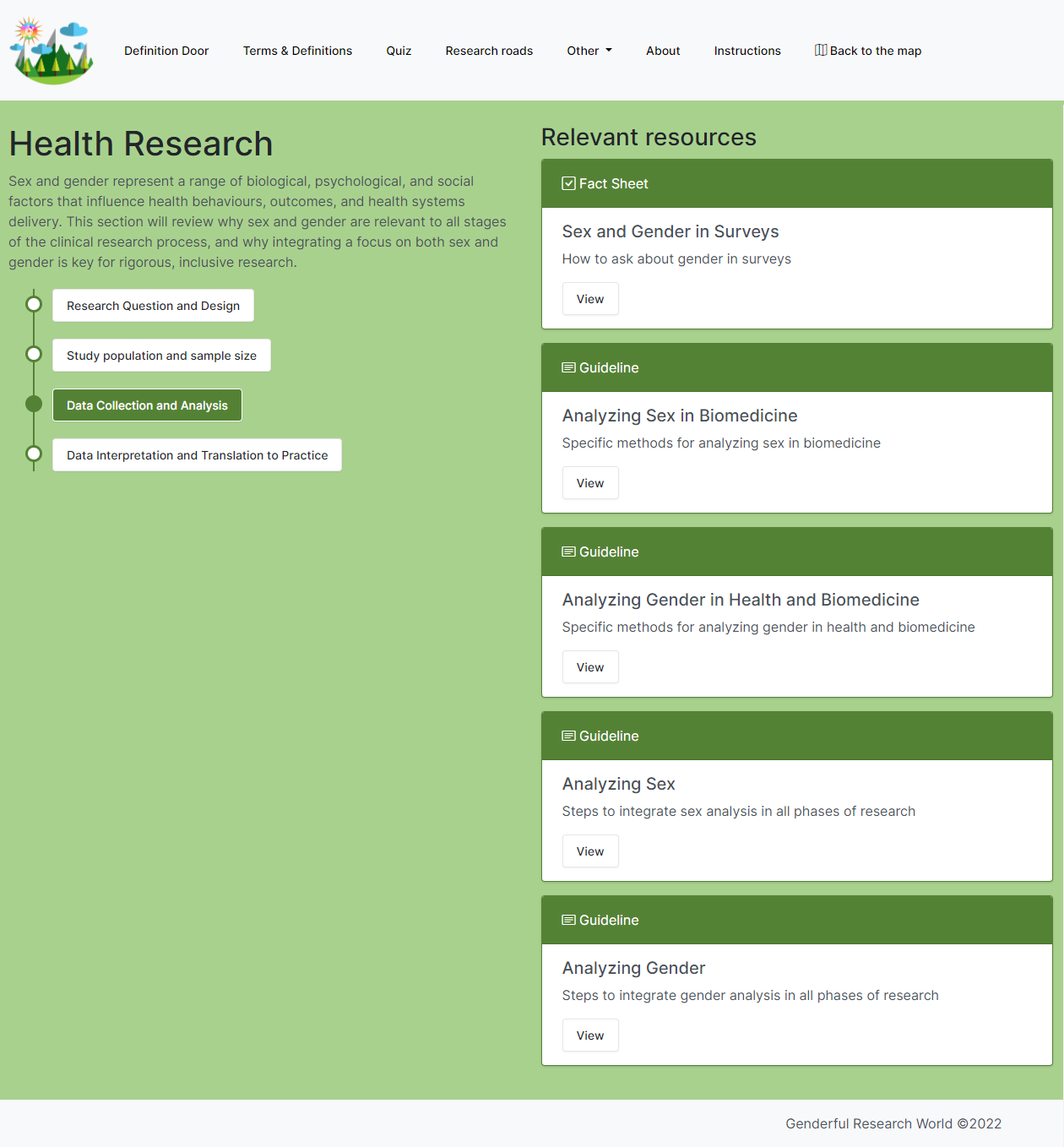

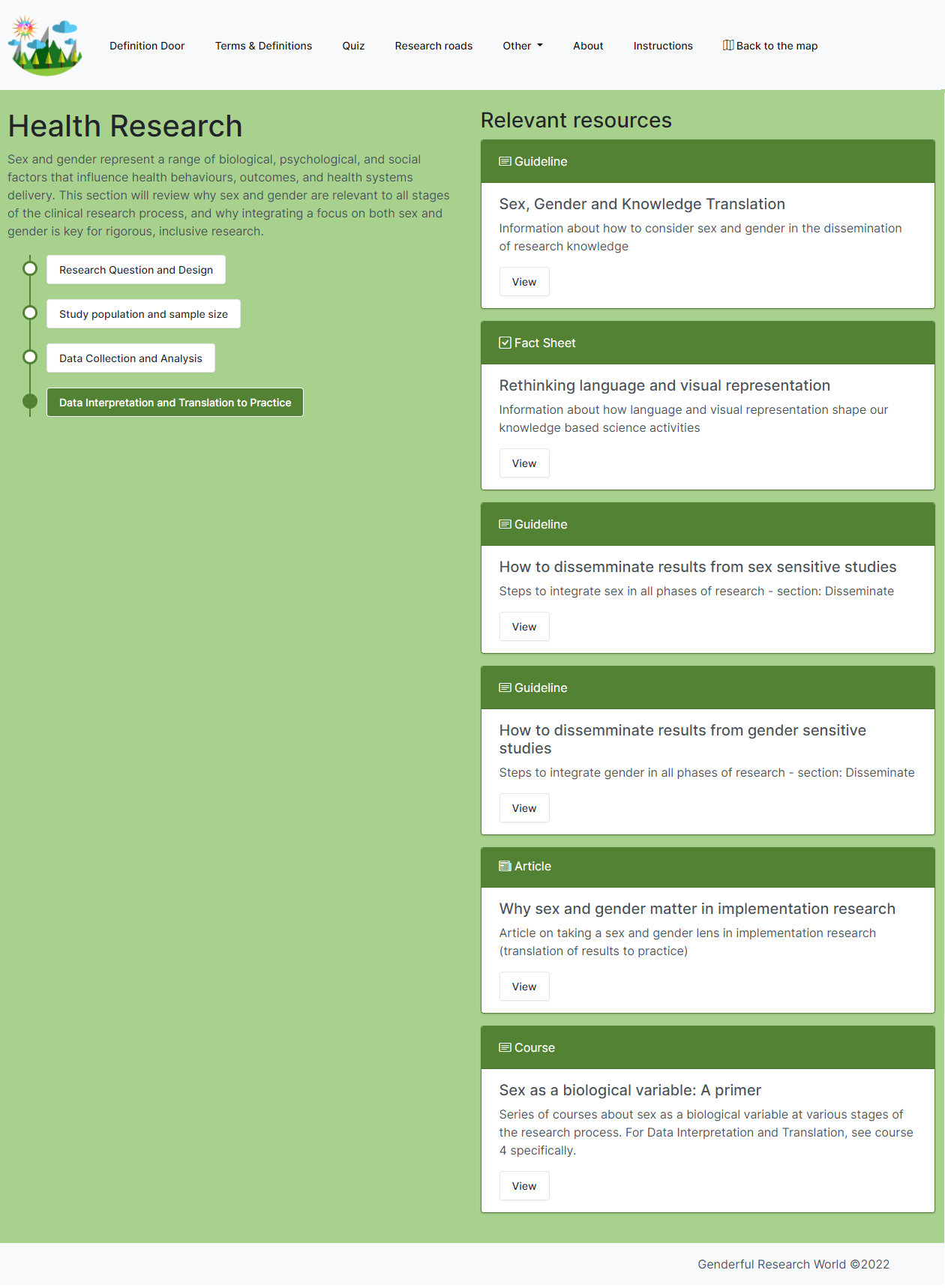


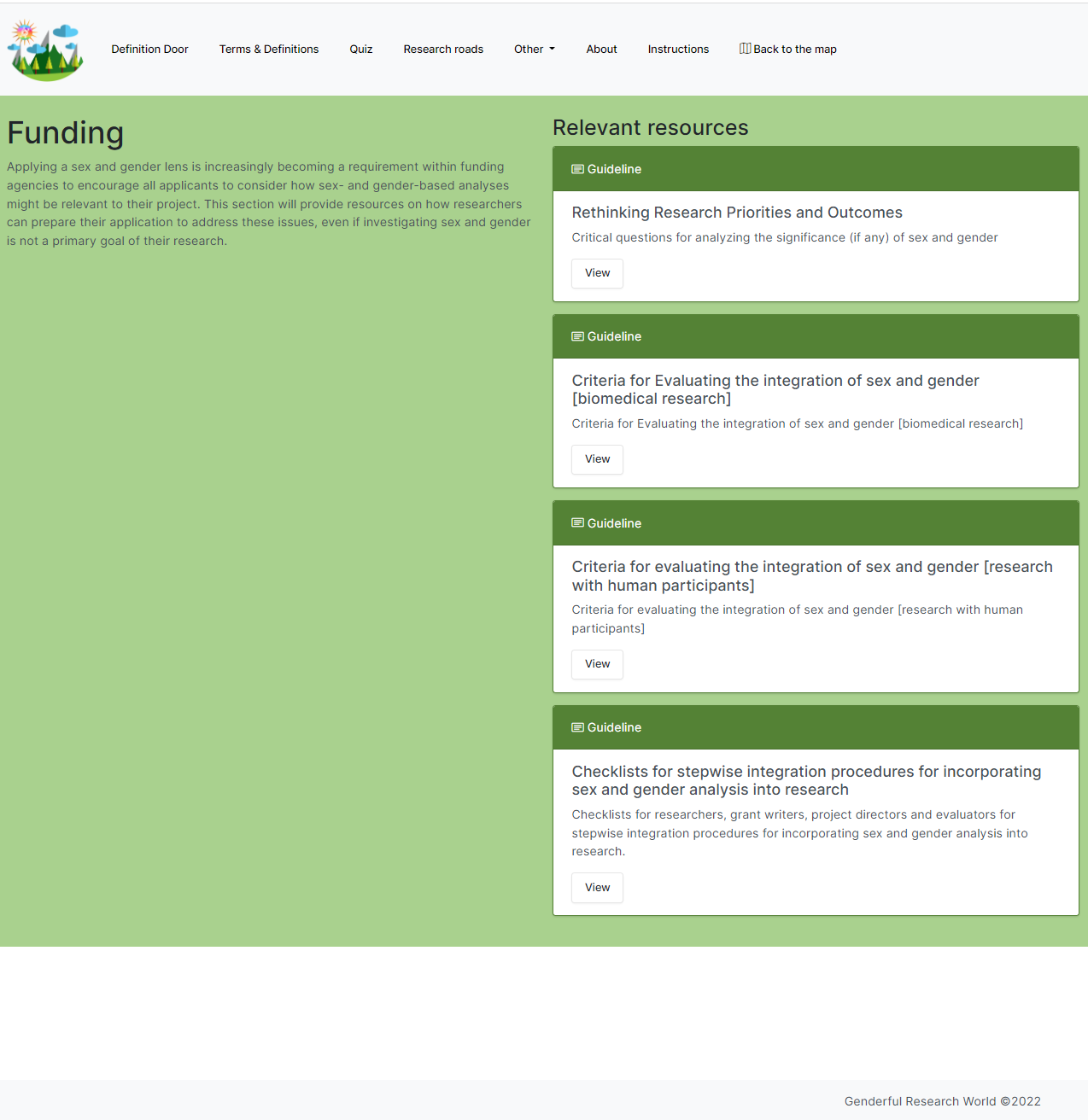

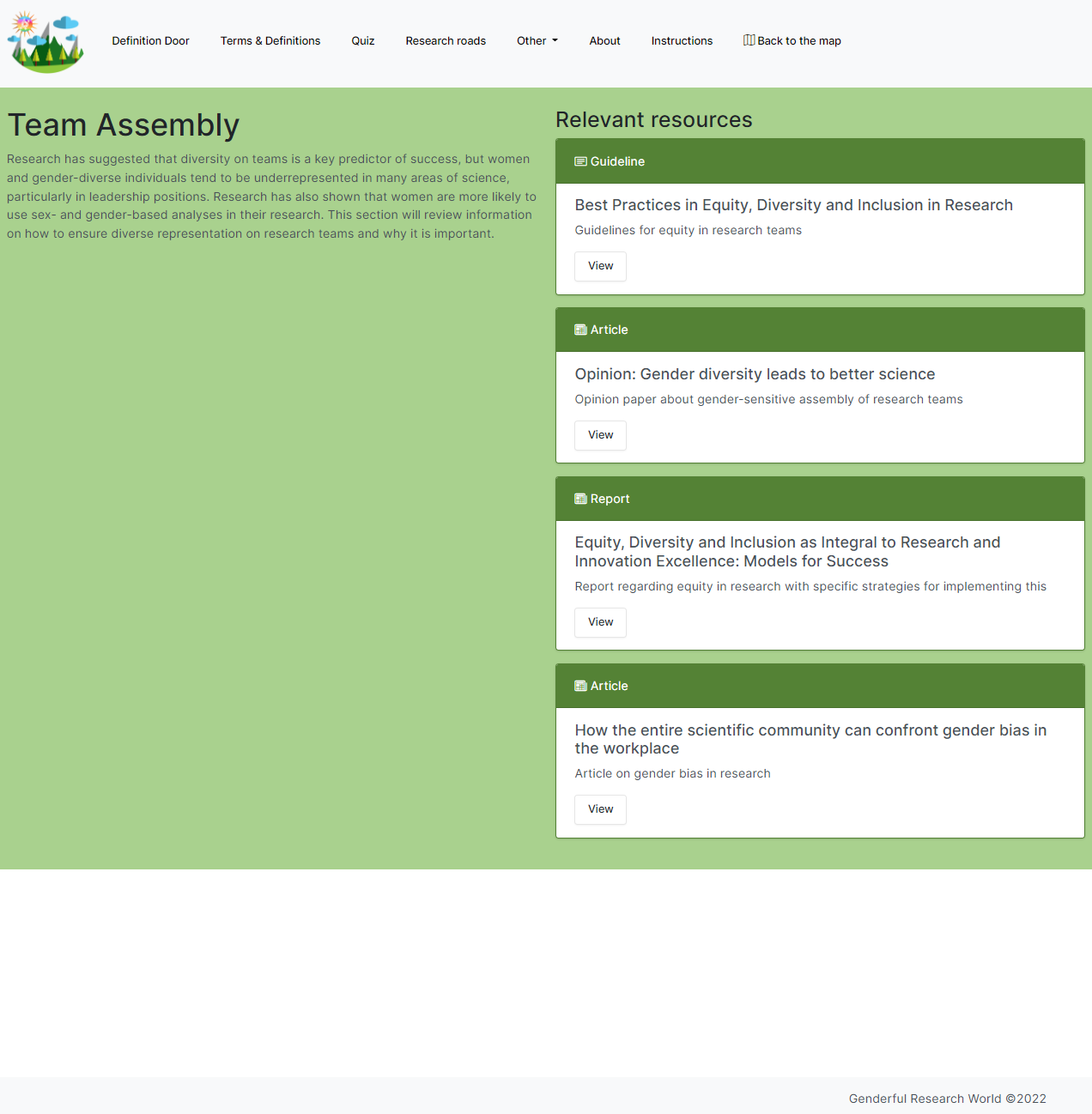

Supplement: Supplementary file 1 — Supplementary Material 1 [file 12939_2023_1899_MOESM1_ESM.docx]
