# Supplementary material for: A genderful research world: rapid review, design, and pilot study of an interactive platform for curated sex and gender health research resources
Source: Int J Equity Health. 2023 Jun 20;22:118. doi: 10.1186/s12939-023-01899-2 (PMC10283329; doi:10.1186/s12939-023-01899-2)
Supplement: Supplementary file 2 — Supplementary Material 2 [file 12939_2023_1899_MOESM2_ESM.docx]

# **Appendix A: Rapid review protocol**

**1** **Research Title**

Developing an interactive sex and gender resource platform for biomedical and health scientists: a rapid review of resources.

**2** **Review Objectives**

o To collect key sex and gender resources for PhD and Post-doc pre-clinical and clinical health researchers who are new to sex and gender sensitive research and want to integrate sex and gender dimensions into their research for the following research stages and phases:

§ Introduction into sex and gender terminology and relevance for biomedical and health research.

§ Sex and gender integration in assembling research teams.

§ Sex and gender integration in research question and study design.

§ Sex and gender integration in study population and sample size.

§ Sex and gender integration in data collection and follow-up (if applicable).

§ Sex and gender integration in data analysis.

§ Sex and gender integration in interpretation and translation to medical and health practice.

§ Sex and gender integration when applying for funding

**3** **Types of resources to be included**

Criteria for inclusion are sex and gender in health research resources that:

Primary

1. Affiliated with the EU Gendered Innovations project

2. Affiliated with the Canadian Institute of Health Research Institute of Gender and Health

Secondary:

1. Provide key information for the research stages and phases specified in the review objective, which cannot be identified through the primary inclusion criteria. E.g resources that are:

a. ~~Affiliated with the Sex and Gender Specific Health project~~ (Note: the decision was made after the commencement of the rapid review to remove this specific criteria).

b. Affiliated with sex and gender consortia related to biomedical and/or health research.

Resources will be excluded when:

a. They have been published before 2015 and/or have not been updated since before 2015.

b. They are not available in English.

c. They are not publicly accessible via the internet.

**4** **Database Searches**

Primarily, the databases of the EU Gendered Innovations initiative and Canadian Institute for Health Research will be searched page by page for key resources. We’ve selected these project databases as starting point because they include multiple valuable resources and have been recognized as projects driven by expert groups in sex and gender sensitive medicine. Additionally, hand searches will be performed to fill missing resource gaps that emerge during the review process.

**5** **Condition or Domain being studied**

Identify essential resources for integrated sex and gender in pre-clinical and clinical health research.

**6** **Participant/Population**

N.A.

**7** **Intervention(s), Exposure(s)**

N.A.

**8** **Comparator(s)/control**

N.A.

**9** **Context**

Pre-clinical and clinical health research.

**10** **Main outcome(s)**

Main outcomes from this rapid review will be an overview of:

1) 5-7 key resources per research stage or phase for pre-clinical and clinical health research.

**11** **Data extraction (selection and coding)**

First, the websites of the EU Gendered Innovation and the Canadian Institute of Health Research will be screened page by page for resources related to the formulated research stages for this review. Relevant resources will be extracted and labeled according to the following information: 1) Relevance for which research phase, 2) Objective of the resource, 3) Applicable to pre-clinical and/or clinical research, 4) Type of resource.

Secondly, if key resources are missing or their availability is limited through the abovementioned project-sites, hand searches will be performed to fill the information gap as identified during the review process. Searches will continue until 5-7 relevant resources per research phase have been identified and/or until the screening deadline of September 1^st^ 2021.

**12** **Risk of bias (quality) assessment**

N.A.

**13** **Strategy of data synthesis**

An overview will be developed; it will summarize the identified resources. Subsequently, 5-7 key resources will be selected per research phase that will be used for the development of the Genderful Research World interactive platform. This selection will be made based on peer-discussions between research group members.

**14** **Analysis of subgroups or subsets**

N.A.

**Type and method of review**

Rapid review

**Anticipated or actual start date**

01.08.2021

**Anticipated completion date**

15.09.2021

**Funding sources/sponsors**

This project is funded by ZonMw and CIHR-IGH.

**Conflict of interest**

None to declare

**Language**

English

**Country**

International

**Subject index terms**

Sex and Gender Resources
