# Supplementary material for: A genderful research world: rapid review, design, and pilot study of an interactive platform for curated sex and gender health research resources
Source: Int J Equity Health. 2023 Jun 20;22:118. doi: 10.1186/s12939-023-01899-2 (PMC10283329; doi:10.1186/s12939-023-01899-2)
Supplement: Supplementary file 4 — Supplementary Material 4 [file 12939_2023_1899_MOESM4_ESM.docx]

Appendix D: Demographics, applicability, usability, and desirability surveys

**Demographics questionnaire**

Age  years

Sex  male  female

intersex

Gender identity  man  woman

non-binary  other, namely: ………..

Country of origin ………………………………………………………………

Country of residence ………………………………………………………………

Career level  Junior/PhD level researcher
  Mid-level/Postdoc researcher
  Senior researcher /(Ass.) Professor

Research field  preclinical/basic

clinical

public health

Research area of expertise/interest ……………………………………………………………….

What information would you be most interested in or is most relevant to you right now? Information about…

Sex and gender integration in **assembling research team**s
 Sex and gender integration when **applying for funding** Sex and gender in **biomedical research**
 Sex and gender in **health research**

For which research phase(s) would you be most interested in seeing sex and gender resources right now? (*multiple answers possible*)

Funding
 Study population and sample size
 Research question and study design
 Data collection and follow-up
 Data analysis
 Interpretation and translation to medical and health practice
 Whole pipeline

Which device did you use to visit the GRW platform?

Desktop or laptop computer
 Mobile phone

**Applicability questionnaire**

Please select one of the following 5 options for each statement and respond with the first answer that comes to mind. Your answers to this questionnaire are anonymous and voluntary.

1. The quiz triggered my knowledge about sex and/or gender

| Totally disagree | Disagree | Neither | Agree | Totally agree |
| --- | --- | --- | --- | --- |
|  |  |  |  |  |

| Explanation (not mandatory) |
| --- |
|  |

2. The quiz made me think more extensively about sex and gender

3. It was easy to move through the questions from the quiz

4. The organization of the resources on the GRW (i.e. integration of the resources through the research phases) is a good way of finding the right information

5. The research phases integrated in the GRW cover the most important aspects of the research process

6. The resources that the GRW recommended are useful to me

7. The resources recommended by the GRW were new to me

8. The resources recommended by the GRW covered enough information

9. How would you rate the overall applicability of the GRW? (i.e. how relevant it was to you and your needs)

0 1 2 3 4 5 6 7 8 9 10

☹ 😐 ☺

**Usability questionnaire**

1. Access to the GRW was quick and intuitive

| Totally disagree | Disagree | Neither | Agree | Totally agree |
| --- | --- | --- | --- | --- |
|  |  |  |  |  |

| Explanation |
| --- |
|  |

2. The GRW website was easy on the eyes (e.g. font, font size, image quality)

3. The instructions at the beginning the GRW were clear enough so I could understand how to move through the website

4. I quickly found the information I was looking for on GRW

5. I became familiar with the GRW quickly

6. The GRW was easy to use (no extra skills needed, not too complex)

7. The various functions (e.g. quiz, interactive road map, tabs) were well integrated in the GRW

8. The information text on the GRW was clear

9. The information text on the GRW was appropriate

10. Terminology used on the GRW was understandable

11. The application was device-friendly

12. I enjoyed the use of the GRW

13. How would you rate the overall usability of the GRW?

0 1 2 3 4 5 6 7 8 9 10

☹ 😐 ☺

**Desirability questionnaire**

1. The application is innovative

| Totally disagree | Disagree | Neither | Agree | Totally agree |
| --- | --- | --- | --- | --- |
|  |  |  |  |  |

| Explanation |
| --- |
|  |

2. The GRW is designed in an interesting way

3. The information on the GRW was presented in a fun way

4. The aesthetics (visual appeal) improved my experience

5. The GRW is functional for me as a researcher

6. The GRW is a good match with how I prefer to search for resources

7. Use of the GRW is feasible in the time I want to spend

8. The application is useful for researchers at all levels of experience with sex and gender

9. The GRW is a relevant resource for researchers

10. How would you rate the overall added value of the GRW for sex and gender health researchers?

0 1 2 3 4 5 6 7 8 9 10

☹ 😐 ☺

**Open questions**

1. What elements of the GRW did you like?

|  |
| --- |

2. What elements of the GRW could be improved?

|  |
| --- |

3. Did you experience any technical difficulties? (e.g. page didn’t load properly, formatting looked strange on my device, etc.)

|  |
| --- |

4. Would you recommend this application to your research colleagues?

0 1 2 3 4 5 6 7 8 9 10

☹ 😐 ☺
